# Beyond the vaccine baseline: Optimizing mRNA stability and translation via regulatory element engineering

**DOI:** 10.1016/j.omtn.2026.102879

**Published:** 2026-03-15

**Authors:** Stefano Cagnin

**Affiliations:** 1 Department of Biology, University of Padova, 35131 Padova, Italy; 2 CIR-Myo Myology Center, University of Padova, 35131 Padova, Italy

## Main text

The global deployment of messenger RNA (mRNA) vaccines against COVID-19 constituted a pivotal milestone in the history of RNA-based medicine. These achievements were built upon decades of foundational research that demonstrated three critical capabilities: first, the efficient encapsulation of large mRNA transcripts within lipid nanoparticles (LNPs) to facilitate cellular uptake and protect them against extracellular RNases; second, the proof of concept that exogenous mRNA injected into skeletal muscle can successfully hijack host translational machinery to produce functional proteins; and third, the strategic use of modified nucleosides, such as methylpseudouridine, to suppress the innate immune system’s recognition of synthetic RNA through Toll-like receptor 7 and 8 (TLR7 and TLR8) pathways.^1^ Despite this success, the broader landscape of nucleic acid-based therapies remains disproportionately dominated by oligonucleotides. Prior and after the COVID-19 pandemic, regulatory approvals by the FDA (Food and Drug Administration) and EMA (European Medicines Agency) were largely restricted to antisense oligonucleotides (ASOs) and small interfering RNAs (siRNAs).^2^ These molecules possess inherent advantages over mRNA, including greater chemical stability, simplified manufacturing processes, and the ability to be directly conjugated to targeting moieties like N-acetylgalactosamine (GalNAc) for hepatic delivery.^3^ mRNA, by comparison, is a large, fragile macromolecule that requires sophisticated delivery systems and precise engineering of its non-coding regions to ensure therapeutic efficacy.

**Figure 1:**
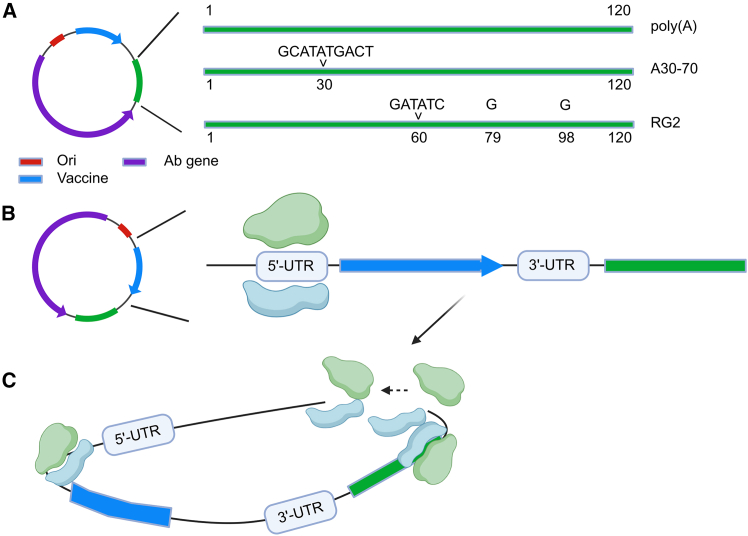
Synergistic optimization of synthetic mRNA regulatory elements (A) Schematic representation of poly(A) tail variants. While continuous A120 tracts are prone to contraction during bacterial propagation, the A30-L-A70 variant incorporates the sequence GCATATGACT after the 30^th^ adenine, and RG2 variant incorporates GATATC sequence after the 60^th^ adenine and strategic guanosine spacers that ensure 100% transmission stability in *E. coli*. (B) UTR engineering strategy. The combination of 5′ UTR and 3′ UTR increases the translational load (polysome formation), maximizing protein output per transcript. (C) The functional closed-loop model. The physical bridge between the optimized 5′ and 3′ ends facilitates efficient ribosome recycling, leading to the sustained and high-level protein expression required for next-generation mRNA therapeutics. Ori, replication origin; Ab gene, antibiotic gene; Vaccine, gene for the synthesis of the mRNA used for the vaccination. Image produced with Biorender.

A critical component of mRNA stability and translation is the poly(A) tail, which protects the transcript from 3′ to 5′ exonucleolysis and recruits poly(A)-binding proteins (PABPs) to initiate translation. However, the production of mRNA containing long, continuous poly(A) tracts presents industrial challenges. In the standard manufacturing workflow, mRNA is transcribed *in vitro* from a linearized plasmid DNA template. These templates are typically propagated in *E. coli*, a host that is notoriously intolerant of long homopolymeric sequences. Genetic recombination within the bacteria often leads to poly(A) tail shrinkage, where the essential adenosine repeat is truncated.^4^ This results in a heterogeneous drug product that fails to meet strict regulatory standards for pharmaceutical consistency and potency. To address this, the field has moved toward segmented poly(A) tails. By introducing non-adenosine linkers, researchers can stabilize the plasmid in bacteria without compromising the mRNA’s biological function. For several years, the “A30-L-A70” architecture (30 adenosines followed by a 10-nucleotide linker and 70 adenosines) has been regarded as the gold standard for stabilizing mRNA therapeutics during scale-up^5^ (Figure 1A). However, as the field moves beyond vaccines toward protein replacement therapies, where protein must be produced at high levels for extended periods, this standard is being re-evaluated. In a significant advancement published in this issue of *Molecular Therapy Nucleic Acids*, Chen and colleagues evaluated the transmission stability and *in vivo* performance of various poly(A) designs.^6^ Their search led to the identification of the poly(A) RG2 variant. This architecture mimics the structure of the traditional segmented poly(A) tail but incorporates a novel design: two guanosine (G) spacers within the second adenosine segment (Figure 1A). RG2 has demonstrated stable bacterial transmission, ensuring its potential for scaling up to the industrial level. More importantly, it not only demonstrated good performance in bacterial transmission avoiding recombination effects but also confirmed its biological potency. In animal models, RG2-modified mRNA supported significantly higher protein expression than previous industry standards. Remarkably, this variant showed no translational variability regardless of the genetic load, the length, or complexity of the coding sequence.^6^ This suggests that it could serve as a universal scaffold for diverse mRNA therapeutics, from small antigens to large therapeutic enzymes.

While the poly(A) tail governs the lifetime of mRNA, the 5′ and 3′ untranslated regions (UTRs) drive translational efficiency. The work of Medjmedj and colleagues provides a crucial complement to the findings on poly(A) architecture. By screening a library of endogenous and synthetic UTRs, they identified specific sequences to use in the UTRs and evidenced that the sequence of the coding region may interfere with the folding of the 5′ UTR and 3′ UTR, leading to conformation that the mRNA is less favorable for translation, with a dramatic reduction in protein output.^7^ Their optimized UTRs function by increasing the translational load, essentially making the mRNA more attractive to ribosomes (Figure 1B). In the competitive environment of the cytoplasm, where synthetic mRNA must compete with endogenous transcripts for limited ribosomal resources, these UTRs provide a decisive advantage. The integration of such high-efficiency UTRs with stable poly(A) variants like RG2 represents the next logical step in mRNA engineering.

Chen and colleagues reported that while 3′ UTR sequences did not significantly impact the bacterial transmission of the RG2 variant, external environmental factors, such as the growth temperature of the bacterial culture, played a definitive role in maintaining the integrity of the poly(A) tract.^6^ This highlights the need for a holistic manufacturing strategy where genetic design and bioprocess parameters are optimized in tandem.

Furthermore, the interaction between the poly(A) tail and the 3′ UTR is biologically fundamental. In nature, the 3′ UTR is the site where microRNAs and RNA-binding proteins (RBPs) dock to regulate the transcript’s fate.^8^ These interactions can either accelerate the removal of the poly(A) tail (deadenylation) or protect it. For example, the closed-loop configuration of mRNA, where the 5′ cap-binding complex interacts with the PABP via the eIF4G scaffold protein, is the primary mechanism for ribosome recycling^9^ (Figure 1C). The efficiency of this loop is directly influenced by the sequence motifs embedded within the UTRs. In many pathological conditions, the cell alters its expression of 3′ UTR isoforms, a process known as alternative polyadenylation. This can lead to transcripts with different stabilities and subcellular localizations, even if the coding sequence remains identical.^10^ For mRNA-based therapy to reach its full potential, especially in treating chronic genetic diseases, we must move toward context-specific design. This would involve selecting UTR and poly(A) combinations that are tailored to the specific cellular environment of the target tissue, whether it be the liver, the heart, or skeletal muscle.

In conclusion, the improvements discussed here, exemplified by the RG2 poly(A) variant and high-performance UTRs, provide a roadmap for increasing the potency of synthetic mRNA. By maximizing the amount of protein produced per molecule of RNA, we can significantly reduce the required therapeutic dose. Lowering the dose is perhaps the most effective way to minimize the risk of LNP-related toxicity and immune system activation and to reduce the cost of products for these life-saving therapies. As we move forward, the focus must remain on the synergy between stability and expression. The untranslated regions of mRNA are no longer an afterthought; they are the primary elements we must turn to in order to fine-tune the genetic medicines of the future. A deeper understanding of these regulatory mechanisms, in both healthy and diseased states, will not only elucidate the fundamental rules of gene expression regulation but will also drive the development of more durable, effective, and safer mRNA-based therapies.

## Acknowledgments

We acknowledge the support provided by the 10.13039/100000002National Institutes of Health (NIH) under grant no. 1R21NS139044-01 and the Research Projects of Relevant National Interest (2022NBJNT).

## Author contributions

S.C. wrote the manuscript.

## Declaration of interests

The author declares no competing interests.
